# Motor-Sparing Effect of Adductor Canal Block for Knee Analgesia: An Updated Review and a Subgroup Analysis of Randomized Controlled Trials Based on a Corrected Classification System

**DOI:** 10.3390/healthcare11020210

**Published:** 2023-01-10

**Authors:** Yu-Hsuan Fan Chiang, Ming-Tse Wang, Shun-Ming Chan, Se-Yi Chen, Man-Ling Wang, Jin-De Hou, Hsiao-Chien Tsai, Jui-An Lin

**Affiliations:** 1 Department of Anesthesiology, Taipei Medical University Hospital, Taipei 11031, Taiwan; 2 Department of Anesthesiology, Tri-Service General Hospital and National Defense Medical Center, Taipei 11490, Taiwan; 3 Department of Neurosurgery, Chung-Shan Medical University Hospital, Taichung 40201, Taiwan; 4 School of Medicine, Chung-Shan Medical University, Taichung 40201, Taiwan; 5 Department of Anesthesiology, National Taiwan University Hospital, Taipei 100225, Taiwan; 6 Division of Anesthesiology, Hualien Armed Forces General Hospital, Hualien 97144, Taiwan; 7 Department of Anesthesiology, School of Medicine, National Defense Medical Center, Taipei 11490, Taiwan; 8 Dianthus MFM Clinic Taoyuan, Dianthus MFM Center, Taoyuan 33083, Taiwan; 9 Department of Anesthesiology, School of Medicine, College of Medicine, Taipei Medical University, Taipei 110, Taiwan; 10 Center for Regional Anesthesia and Pain Medicine, Chung Shan Medical University Hospital, Taichung 40201, Taiwan; 11 Department of Anesthesiology, School of Medicine, Chung Shan Medical University, Taichung 40201, Taiwan; 12 Department of Anesthesiology, Chung Shan Medical University Hospital, Taichung 40201, Taiwan

**Keywords:** analgesia, classification, knee, motor activity: motor-sparing, nerve block: adductor canal block, randomized controlled trial

## Abstract

Objective: Discrepancies in the definition of adductor canal block (ACB) lead to inconsistent results. To investigate the actual analgesic and motor-sparing effects of ACB by anatomically defining femoral triangle block (FTB), proximal ACB (p-ACB), and distal ACB (d-ACB), we re-classified the previously claimed ACB approaches according to the ultrasound findings or descriptions in the corresponding published articles. A meta-analysis with subsequent subgroup analyses based on these corrected results was performed to examine the true impact of ACB on its analgesic effect and motor function (quadriceps muscle strength or mobilization ability). An optimal ACB technique was also suggested based on an updated review of evidence and ultrasound anatomy. Materials and Methods: We systematically searched studies describing the use of ACB for knee surgery. Cochrane Library, PubMed, Web of Science, and Embase were searched with the exclusion of non-English articles from inception to 28 February 2022. The motor-sparing and analgesic aspects in true ACB were evaluated using meta-analyses with subsequent subgroup analyses according to the corrected classification system. Results: The meta-analysis includes 19 randomized controlled trials. Compared with the femoral nerve block group, the quadriceps muscle strength (standardized mean difference (SMD) = 0.33, 95%-CI [0.01; 0.65]) and mobilization ability (SMD = −22.44, 95%-CI [−35.37; −9.51]) are more preserved in the mixed ACB group at 24 h after knee surgery. Compared with the true ACB group, the FTB group (SMD = 5.59, 95%-CI [3.44; 8.46]) has a significantly decreased mobilization ability at 24 h after knee surgery. Conclusion: By using the corrected classification system, we proved the motor-sparing effect of true ACB compared to FTB. According to the updated ultrasound anatomy, we suggested proximal ACB to be the analgesic technique of choice for knee surgery. Although a single-shot ACB is limited in duration, it remains the candidate of the analgesic standard for knee surgery on postoperative day 1 or 2 because it induces analgesia with less motor involvement in the era of multimodal analgesia. Furthermore, data from the corrected classification system may provide the basis for future research.

## 1. Introduction

Femoral nerve block (FNB) has previously been the mainstay for postoperative analgesia following knee surgery for years [1]. However, quadriceps weakness, which is unfavorable for rehabilitation and might delay early ambulation, is a major concern of FNB [2]. Recently, an alternative, adductor canal block (ACB), has been introduced as a motor-sparing nerve block for knee surgery and gained attention from anesthesiologists and orthopedists. ACB has been claimed to provide adequate knee analgesia mainly by blocking the saphenous nerve (SN). As such, it could provide some benefits, including improved mobility and reduced risk of falls [3,4,5,6,7,8,9].

Previously, SN block approached with surface landmarks has been established for analgesia for procedures below the knee [10]. Because of its small size and the lack of a motor component in the SN, conventional SN block has inconsistent success. Then, it has evolved to ultrasound guidance to aid in precision and avoid unintentional puncture of major vessels [11,12]. Manickam et al. described the ultrasound-guided SN block in the adductor canal with the needle entry point at 2–3 cm proximal to the adductor hiatus [13]. Lund et al. introduced ACB as halfway between the iliac spine and the patella [8]. Until now, there have been debates about the nomenclature and techniques regarding ACB.

Recently, there has been growing evidence indicating that ACB is as effective as FNB in providing postoperative analgesia following knee surgery. Many studies revealed that ACB could preserve better quadriceps strength and improve mobilization ability without compromising pain control [8,14,15]. ACB also reduced the risk of falls compared with FNB [16,17,18]. However, other studies demonstrated that FNB had denser analgesia, less analgesic requirement, and greater satisfaction compared with ACB [19]. Additionally, there was no significant difference in quadriceps strength deficits after FNB or ACB [20,21,22].

These discrepancies might result from the different approaches of ACB, or more precisely, from the non-unified definition of ACB. Landmark guidance might risk injection outside the anatomical adductor canal. While searching for studies on ACB, there are many ways to perform ACB according to the injection site, including anatomically defined femoral triangle (FT) and proximal or distal adductor canal [23,24,25,26,27]. In several studies, ACB has been performed at the midpoint of the thigh (the midpoint between the anterior superior iliac spine and the base of the patella) [8,9,15,28,29,30,31,32,33,34,35]. However, some researchers have challenged this definition because the midpoint of the thigh most likely falls within the FT [36]. Although the FT is connected to the adductor canal, the nerves to block differ between these two structures, which may cause clinically meaningful differences [37,38,39]. It is reasonable that the block level can affect the analgesic outcomes, mobilization, and motor-sparing effect. Hence, anatomically classifying published ACB studies before comparing the analgesic effects or other outcomes among different approaches is important. In this study, we will describe the relevant anatomy of ACB, clarify the definition of ACB, develop a nomenclature system to include various approaches, investigate the analgesic and motor-sparing effects by meta-analysis and subgroup analyses based on this corrected classification, and detail the possible complications.

### 1.1. Applied Anatomy

**Figure 1:**
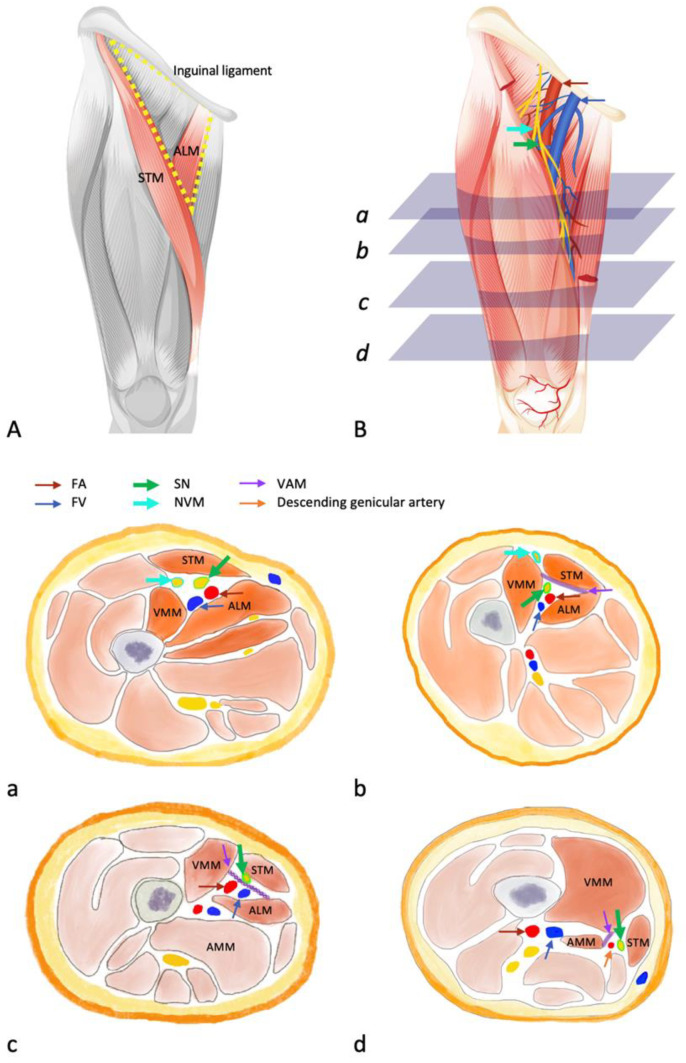
Anatomical illustration of the femoral triangle (FT) and the adductor canal (AC). FA, femoral artery (red arrow); FV, femoral vein (blue arrow); STM, sartorius muscle; ALM, adductor longus muscle; AMM, adductor magnus muscle; VMM, vastus medialis muscle; VAM, vastoadductor membrane (purple arrow); NVM, nerve to vastus medialis (cyanide arrow); SN, saphenous nerve (green arrow); FT, femoral triangle; AC, adductor canal; descending genicular artery (orange arrow).

The relevant anatomy is shown in Figure 1. A comprehensive understanding of the anatomy of the FT and adductor canal is necessary to examine the clinical effects of ACB.

(**A**) The boundaries of the FT and the AC. The base, the medial border, and the lateral border of the FT are the inguinal ligament, medial margin of the adductor longus muscle, and the medial margin of the sartorius muscle. The AC extends from the apex of the FT, defined as the intersection between the medial border of the sartorius muscle and the medial border of the adductor longus muscle. The AC ends at the adductor hiatus of the adductor magnus. (**B**). The contents of the FT and the AC. The femoral neurovascular bundle is in the FT at the inguinal ligament level and dives subsartorially at the distal FT level (the level of mid-way between ASIS and the patella base). SN is anterolateral to FA and lies between STM and VMM. NVM is lateral to SN, and also lies between STM and VMM. In the proximal AC, FV is posterior to FA. The femoral vein becomes lateral to the femoral artery in the distal AC. VAM extends from the VMM, under STM to the ALM and AMM. SN is anterolateral to FA in the proximal AC. SN crosses the femoral artery anteriorly from a lateral to medial direction. SN pierces VAM at mid-AC. In distal AC, SN goes medially. It lies above VAM and between STM and AMM.

Cross-sectional anatomy of (**a**). Distal FT (the level of mid-way between ASIS and the patella base). The anterior, medial, and lateral borders are STM, ALM, and VMM. NVM lies between the STM and VMM and lateral to the SN. SN is anterolateral to FA. FV is posterior to FA. (**b**). Proximal AC (p-AC), the anteromedial border is STM, the anterolateral border is the VMM, and the posteromedial border is the ALM. The medial border of STM meets the medial border of ALM. VAM extends from the VMM, under STM to the tendons of ALM. NVM lies outside the AC in a fascial on VMM. SN is antero-lateral to FA. FV is posterior to the femoral artery. (**c**). Mid AC, the ALM is replaced by AMM posteromedially. SN penetrates the VAM and lies superficial to VAM adjacent to the descending genicular artery. (**d**). Distal AC (d-AC), the posteromedial border is AMM. VAM connects the VMM and the AMM. The femoral vessels dive deeply and leaves the AC through adductor hiatus. SN lies above the VAM between the STM and AMM and crosses the AC from the anterior to the medial side.

#### 1.1.1. Femoral Triangle (FT, Scarpa’s Triangle)

The FT is an area in the proximal part of the anterior thigh. The base of the triangle is the inguinal ligament. The medial border is the medial margin of the adductor longus muscle, and the lateral border is the medial margin of the sartorius muscle. The roof is the fascia lata. The floor is formed medially by the adductor longus and the pectineus and laterally by the iliopsoas muscle. The apex of the triangle is continuous with the proximal end of the adductor canal [37,38,40]. The FT contains neurovascular structures, from lateral to medial, including the femoral nerve, femoral vessels, and lymphatics. The femoral neurovascular bundle is in the FT at the inguinal ligament level and dives subsartorially at the distal FT level (the level of mid-way between ASIS and the patella base) [41].

The femoral nerve divides into branches in the FT. The motor branches supply the quadriceps femoris and the hip flexor, whereas the sensory branches innervate the anterior and medial aspect of the thigh (the medial and intermediate cutaneous nerve of thigh), the anteromedial side of the knee (SN, nerve to vastus medialis (NVM)), and the medial side of the leg and foot (SN) [42]. At the distal FT level, the SN and NVM lie subsartorially and outside the FT [41].

#### 1.1.2. Adductor Canal (Hunter’s Canal)

The adductor canal (AC) is an aponeurotic tunnel in the thigh, deep to the sartorius, and serves as a passageway that allows structures to move from the FT to the popliteal fossa. It extends from the apex of the FT, defined as the intersection between the medial border of the sartorius muscle and the medial border of the adductor longus muscle, to the adductor hiatus of the adductor magnus. The boundaries of the AC are the sartorius anteromedially, the vastus medialis anterolaterally, and the adductor longus (in the upper part) or the adductor magnus (in the lower part) posteromedially [36,41]. The roof of the entire AC is the vastoadductor membrane (VAM), and the space between the sartorius muscle and VAM is called the subsartorial compartment [36,41,42,43,44,45]. Superficial to the VAM is the subsartorial plexus, supplying the medial part of the knee [41]. The AC is also known as the subsartorial canal or Hunter’s canal [42]. However, the term “subsartorial canal” may cause confusion. Subsartorial space is a space under the sartorius muscle and extends beyond the vastoadductor membrane [41]. The subsartorial space is distinct from the true adductor canal. We should not regard the “subsartorial canal” as the true adductor canal because the term would cause misunderstanding.

In the AC, the nerve without conflict is the SN. The SN enters the AC anterolateral to the femoral artery and crosses the femoral artery anteriorly from the lateral to the medial direction [46]. In the mid-to-distal third of the AC, the SN penetrates the VAM and leaves the canal with the saphenous branch of the descending genicular artery. The SN is a sensory nerve, supplying the anteroinferior and medial aspects of the knee and the medial side of the leg and ankle [11,47].

Other structures, for example, the NVM, the medial cutaneous nerve of the thigh, and the anterior or posterior branch of the obturator nerve have been reported to pass through the AC; however, the precise locations still remain controversial [3,7,36,46,48,49]. In an anatomic study, Burckett et al. reported that the NVM descends superficially to the VAM; other studies found that the NVM lies inside the fascia, covering the vastus medialis outside the AC [41,46,50,51]. The NVM not only gives branches to the vastus medialis but also innervates the anteromedial capsule superior to the patella and the medial retinaculum [46,52,53]. Therefore, the NVM is also a vital nerve to target when treating pain for patients undergoing surgery involving the medial region of the knee and the distal portion of the femur.

#### 1.1.3. Knee Sensory Innervation

The sensory innervation of the knee can be divided into the anterior and posterior groups. In the anterior group, the cutaneous layer is supplied by the medial femoral cutaneous nerve, the infrapatellar branch of the SN medially, and the lateral femoral cutaneous nerve and the common peroneal nerve laterally. The anterior knee joint capsule is supplied by the branches of the NVM, intermedialis, and lateralis. Furthermore, the genicular nerves, common peroneal nerve, and recurrent peroneal nerve terminate in the anterior aspect of the knee capsule [41,53,54].

The posterior group consists of articular branches from the posterior division of the obturator nerve and the tibial nerves, supplying the posterior knee capsule [55].

### 1.2. Corrected Classification System

A study discussing the precise location of the AC, which involved healthy volunteers, was published in 2017 [36]. However, after that, some studies still defined the mid-thigh needle entry point as one of the approaches of ACB. In addition to the inconsistent ACB approaches, the definition of “mid-thigh” also varies; for example, midway between the anterior superior iliac spine (ASIS) and patella, midway between the greater trochanter and patella, and midway between the inguinal ligament and patella. There is still no consensus on its terminology after a rapid increase in the number of studies. The possible reason for the divergent ACB definition is that some studies defined it according to the surface landmark in the early period, and some studies defined it according to sonoanatomy recently (Table 1). Therefore, we provided a corrected classification system with a unified nomenclature to anatomically categorize the various existing ACB approaches into three groups: femoral triangle block (FTB), proximal ACB (p-ACB), and distal ACB (d-ACB). This classification is according to the anatomy of the AC and the FT.

### 1.3. Femoral Triangle Block (FTB)

FTB refers to an injection at the midway between the ASIS and the base of the patella. Its injection site is subsartorial and anterolateral to the femoral artery and proximal to the apex of the FT, which blocks the SN, the NVM, and the medial femoral cutaneous nerve [41].

The advantages of FTB include that local anesthetic administration may be proximal enough to reliably block the SN and NVM and provide a better analgesic effect while minimizing the spread to the popliteal fossa [46]. Its disadvantages include that local anesthetic may spread to the proximal FT and involve the femoral nerve branches, which may cause quadriceps weakness.

During literature research, the ACB technique has been described as an injection at the “mid-thigh” level in many randomized controlled trials (RCTs). As early as 2014, Bendtsen et al. stated that using the term “ACB” with the mid-thigh needle entry point was a misnomer [26,43,57]. In 2017, a study involving healthy volunteers proved that the midpoint of the thigh, defined as halfway between the ASIS and the base of the patella, mostly resides in the FT [36]. The mean distance from the midpoint of the ASIS and patella to the proximal end of the AC is 4.6 cm [27,36,41]. However, there had been other descriptions of the “mid-thigh” level regarding ACB, such as the midpoint between the greater trochanter and patella, or the midpoint between the inguinal ligament and the patella. Most studies had not described the mid-thigh level clearly. Without a unified definition of the mid-thigh level in these studies, whether the block level was in the FT or in the AC cannot be determined. Hence, the results of the existing studies on ACB should be explained carefully.

### 1.4. Proximal ACB (p-ACB)

Instead of using surface landmarks to determine the injection site of ACB, ultrasound guidance is used to better identify the location of the AC. Under ultrasound guidance, the apex of the FT is identified as the intersection of the medial borders of the sartorius muscle and the adductor longus muscle. This apex is the proximal end of the AC. The distal end of the AC is where the femoral artery becomes deep and passes through the AC hiatus on its way to the popliteal fossa [46,58]. p-ACB targets the AC at its entrance just distal to the apex of the FT.

The potential advantages of p-ACB include a comparable analgesic effect with that of FNB or FTB without increasing quadriceps muscle weakness following knee surgery. Some studies have recently discussed the clinical effect of p-ACB with a precise location under ultrasound guidance. We will discuss the clinical efficacy of p-ACB through a systemic review and meta-analysis in the paragraph below.

p-ACB may have a more significant analgesic effect than d-ACB, whereas it also likely preserves quadriceps motor function. This description can be demonstrated by the cadaveric studies that have found that a dye injected into the proximal AC can spread to the SN throughout the AC and the branches of NVM, including the posteromedial branch and intra-articular branches. The posteromedial branches of the NVM, which terminate as the superior medial genicular nerve and the branch to the vastus medialis obliquus, innervate the anteromedial knee joint and muscles. Even though the posteromedial branches of the NVM are involved, the anterior branches of the NVM were not stained, which would likely preserve greater vastus medialis activity, resulting in the quadriceps motor-sparing feature of the ACB [50,58,59]. In contrast, the NVM was barely stained by dye injection in d-ACB because of the two layers of the distal roof of the AC (VAM and the aponeurosis of vastus medialis obliquus). Containing the posteromedial branches of the NVM makes p-ACB a more effective pain control technique for knee surgery [52,59].

### 1.5. Distal ACB (d-ACB)

The point of injection in d-ACB is approximately 1–2 cm proximal to the adductor hiatus. This injectate at the distal part of the AC may spread to the popliteal fossa and block the popliteal plexus, which is formed by the tibial nerve and posterior obturator nerve, innervating the posterior aspect of the knee. A cadaveric study showed that the 10 mL of injection in the distal part of the AC spread into the popliteal fossa [58]. The possibility of local anesthetics spreading to the SN and the popliteal fossa implies that d-ACB provides analgesia to the posterior knee compartment, but also causes foot drop [14,60,61]. Studies comparing different injection locations for ACB (p-ACB and d-ACB) in terms of analgesic and motor-sparing effects will be discussed in the following section (see Section 3.2 The effect of p-ACB versus d-ACB).

### 1.6. ACB Techniques

**Figure 2:**
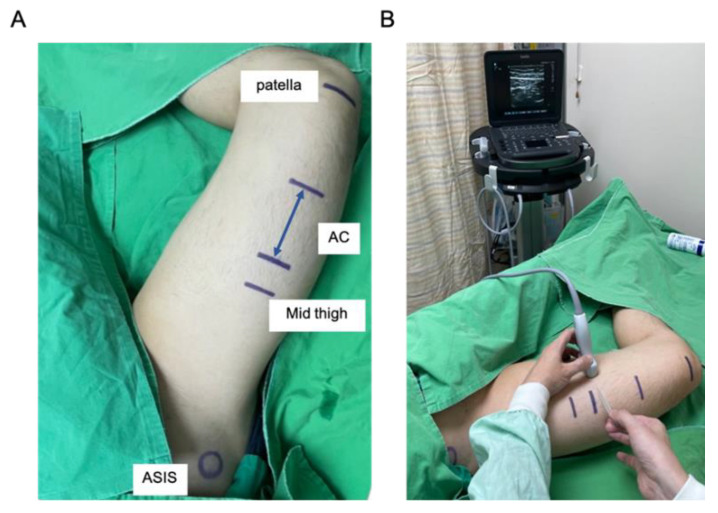
Ergonomic position for proximal adductor canal block. (**A**) Patient position for mid-thigh localization. The patient is in a supine position with the hip externally rotated and the knee slightly flexed. (**B**) Operator and ultrasound machine position. Operators stand same side and lateral to the leg, hold the needle with dominant hand, and position the ultrasound machine on the opposite side of the patient. AC, adductor canal; ASIS, anterior superior iliac spine.

**Figure 3:**
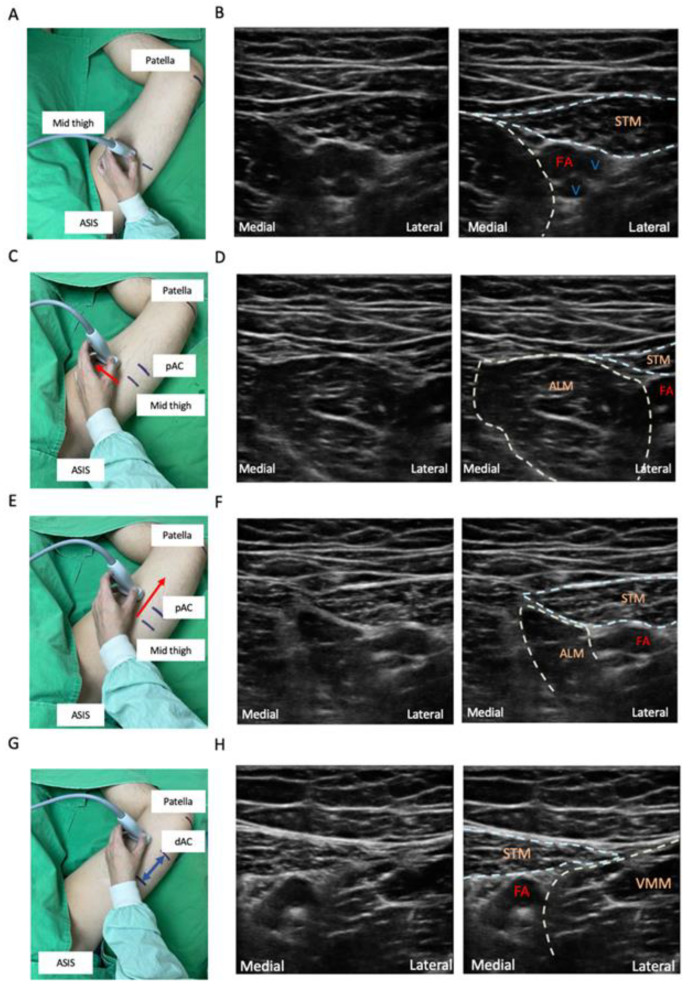
Ultrasound identification of the adductor canal. (**A**) Mark the mid-thigh location midway between the ASIS and base of the patella. Place the probe transversely to obtain short axis view. (**B**) Identify the femoral vessels. Distinguish the artery by compression sign. (**C**) Slide the probe medially (red arrow) to visualize (**D**) the medial borders of the sartorius and adductor longus muscle. (**E**) Slide the probe caudally (red arrow) along the medial borders of the sartorius muscle and the adductor longus muscle to visualize (**F**) the point of the start of the adductor canal, intersection of the medial borders of sartorius muscle and adductor longus muscle. (**G**) Adjust the probe to make the neurovascular bundle on the medial side of the ultrasound screen. Then, slide the probe caudally until (**H**) the femoral artery goes deep to the adductor hiatus. ASIS, anterior superior iliac spine; pAC, proximal adductor canal; dAC, distal adductor canal; FA, femoral artery; V, femoral vein; STM, sartorius muscle; ALM, adductor longus muscle; VMM, vastus medialis muscle.

Ultrasound guidance is now considered the gold standard for peripheral nerve blocks [62]. Ultrasound-guided FTB, p-ACB, and d-ACB were described according to the unified nomenclature systems. Usually, a high-frequency linear transducer is adequate for most ACB or FTB. To optimize sonoanatomy visualization, appropriately rotating and tilting the probe and adequate pressure are recommended. Based on the physical ergonomics, keeping the eye, hand, needle, probe, and ultrasound instrument all in the same plane facilitates needle visualization [63]. The patient is placed in the supine position with the hip externally rotated and the knee slightly flexed (Figure 2A). This external rotation can make the SN lie lateral to the femoral artery in the ultrasound image. Thus, the needle trajectory is shortened without passing through the vastus medialis muscle. Operators stand on the same side and lateral to the leg, hold the needle with the dominant hand, and position the ultrasound machine on the opposite side of the patient (Figure 2B) [64]. Following physical ergonomics can protect operators from injuries, improve needle visualization, and reduce the risk of patient injury. After patient positioning, we suggest the scanning steps as follows:

- Mark the location midway between the anterior superior iliac spine (ASIS) and the base of the patella. Place the transducer transversely on the marker of the thigh to obtain a short-axis view. The femoral vessels will be identified beneath the sartorius muscle. The artery can be distinguished by color doppler flow imaging or by compression sign (Figure 3A,B).
- Slide the probe along the medial border of the sartorius muscle to visualize the intersection of the medial borders of the sartorius muscle and the adductor longus muscle. This point means the start of the AC (Figure 3C–F).
- Slide the probe caudally until the femoral artery goes deep into the echogenic adductor magnus tendon and then passes through the adductor hiatus. This point is the end of the AC (Figure 3G,H).

The position of the probe of ultrasound-guided FTB and ACB, and the matching ultrasound images are shown in Figure 3.

### 1.7. Ultrasound-Guided FTB

- After identifying the AC, place the probe halfway between the base of the patella and the ASIS for a transverse view. The superficial femoral artery (SFA) is identified underneath the sartorius muscle. The SN is usually visible as a hyperechoic structure anterolateral to the artery typically [7,65].
- Once the neurovascular bundle is seen, adjust the probe so that the bundle is on the medial side of the ultrasound screen. The needle is placed lateral to the SN and femoral artery using an in-plane technique. A periarterial injection of local anesthetics is performed at this level, involving the SN, NVM, and medial and intermediate femoral cutaneous nerves [41]. It may also affect the motor branches of the femoral nerve [7].

### 1.8. Ultrasound-Guided p-ACB

- At the distal FT, move the probe caudally about 1–2 cm beyond the apex of the FT. At this level, SN can usually be visualized laterally to the SFA with the thigh in external rotation, and injection between the SFA and SN helps achieve a successful ACB with 2–5 mL of local anesthetics.

### 1.9. Ultrasound-Guided d-ACB

As for d-ACB, (7) place the probe in the distal third of the thigh, where the SFA is also seen beneath the sartorius muscle. Then, the artery is identified diving posteriorly through the adductor hiatus when tracking down using the probe. [13] The point of injection of d-ACB is approximately 1–2 cm proximal to the adductor hiatus.

### 1.10. Systematic Review of RCTs on the Divergent Definition of ACB

Systematic review and meta-analyses have compared mixed ACB (including both with and without clear disclosure of the anatomical definition of the AC) with FNB in knee surgery; however, there are still no conclusive results regarding the motor-sparing effects and analgesic effects without applying a corrected classification system. Some studies showed that patients receiving ACB or FNB have similar clinical efficacy, including pain scores and opioid consumption. Meanwhile, patients receiving ACB have better quadriceps strength, mobilization, and ambulation [66,67,68,69,70,71,72,73,74,75]. Some systematic reviews and meta-analyses have compared ACB with FNB in patients undergoing anterior cruciate ligament reconstruction (ACLR), and the results are similar to those shown in total knee arthroplasty (TKA) [76,77]. In contrast, Ramizadeh et al. showed no differences in quadriceps muscle strength between ACB and FNB at 24 h after surgery [19], and three studies have reported no significant difference in quadriceps muscle strength at 48 h after surgery [78,79,80].

Among several meta-analyses comparing ACB with FNB, one of the most important limitations is the lack of a uniform block level among ACB approaches, which may cause considerable heterogeneity and unreliable results. Based on the studies on the location of the AC from Wong et al. [36], the biases observed in previous studies misinterpreting mid-thigh injection as ACB make the motor-sparing effect of “true” ACB to be spuriously underestimated, and the analgesic effect of “true” ACB to be spuriously overestimated.

In this study, we distinguish FTB, p-ACB, and d-ACB by analyzing the block location according to the ultrasound description in the index studies previously claimed by the author to be ACB. Based on this corrected classification system, we reclassified these previously-claimed ACB approaches into FTB, p-ACB, and d-ACB, and conducted a meta-analysis to survey the clinical effect of these three techniques. Regarding the fact that the injection site of FTB is proximal to true ACB, and it may involve the medial femoral cutaneous nerve, SN, and NVM, our hypotheses are as follows: (1) the motor-sparing effect of “mixed ACB with the exclusion of FTB” is more significant than that of FNB; (2) d-ACB may have less analgesic effect than p-ACB; (3) true ACB provides quadriceps motor-sparing effect and less analgesic effect than FTB.

## 2. Materials and Methods

Our systematic review was registered with PROSPERO, the international prospective register of systematic reviews of the National Institute for Health Research (accessed on 30 October 2020. CRD42020219432). This study followed the Preferred Reporting Items for Systemic Reviews and Meta-analyses guidelines [81].

### 2.1. Search Strategy

Databases including the Cochrane Library, PubMed, Web of Science, and Embase, were searched using the following terms: “Adductor canal” or “Adductor canal block” to identify potentially eligible studies evaluating the efficacy of ACB versus other analgesia methods in patients undergoing knee surgery until February 2022 with the exclusion of non-English studies.

### 2.2. Inclusion and Exclusion Criteria

Inclusion criteria:

(1) Study type: clinical RCTs
(2) Subjects: patients who underwent knee surgeries without limitations
(3) Interventions: comparison of ACB with other methods for knee surgeries, including local infiltration, periarticular local anesthetic infiltration, FNB, interspace between the popliteal artery and the capsule of the posterior knee block, and epidural analgesia, after knee surgery.

Any disagreement in study selection was discussed by two reviewers or arbitrated by a third reviewer. Exclusion criteria:

(1) Non-English articles
(2) No full text available

Risk and bias were assessed using the Cochrane Collaboration tool [82]. We used a random-effects model to represent between-studies heterogeneity. Software of Review Manager (RevMan, version 5.4; The Nordic Cochrane Center, The Cochrane Collaboration, Copenhagen, Denmark) was used for meta-analysis.

## 3. Results

**Figure 4:**
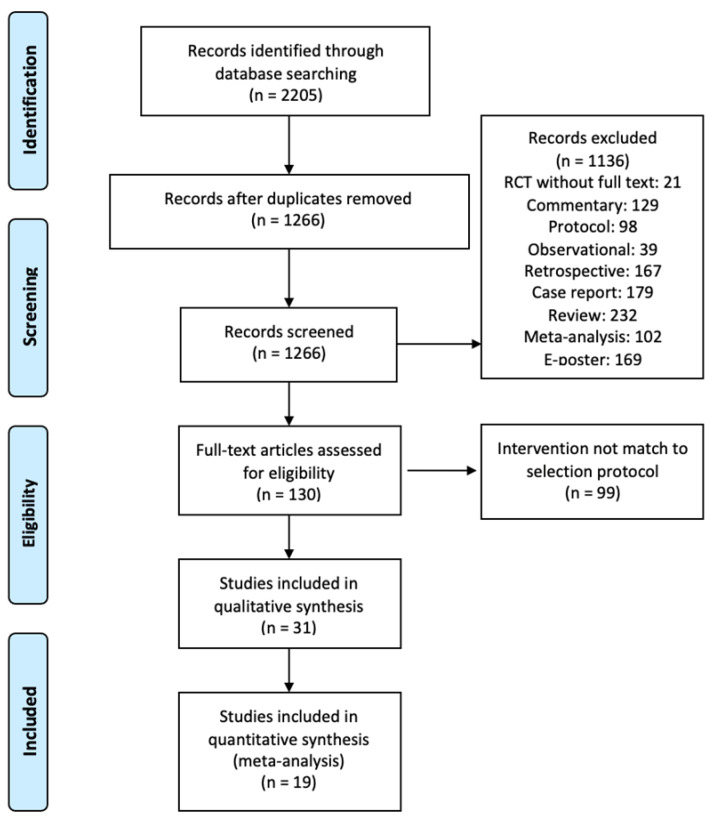
The research results and selection procedure.

Through our systematic database research, 1266 records (after eliminating duplicates) were identified (Figure 4). After removing the studies that did not meet the inclusion criteria, we reviewed 130 full-text articles and found that 66 studies used an unclear or inconsistent approach to ACB (Table 1). The variants of ACB intervention were described according to the surface landmark as “halfway between the ASIS and patella” in 27 RCTs, “halfway between the greater trochanter and the patella” in four RCTs, “halfway between the inguinal ligament and the patella” in 12 RCTs, and “halfway between the inguinal ligament and the medial condyle” in two RCTs. According to ultrasound images, ACB interventions were described as follows: “SFA covered by the medial border of the sartorius muscle” in five RCTs, “FA covered by the midpoint of the sartorius muscle” in four RCTs, “FA underneath the sartorius muscles” in 10 RCTs, and “cavity surrounded by the medial femoris, ALM, and sartorius muscle” in five RCTs.

As shown in Table 2, we checked the original grouping of the published literature and examined whether they should be anatomically re-classified as FTB, p-ACB, or d-ACB in these 66 reported studies.

### 3.1. The Clinical Effect of Mixed ACB versus FNB

**Figure 5:**
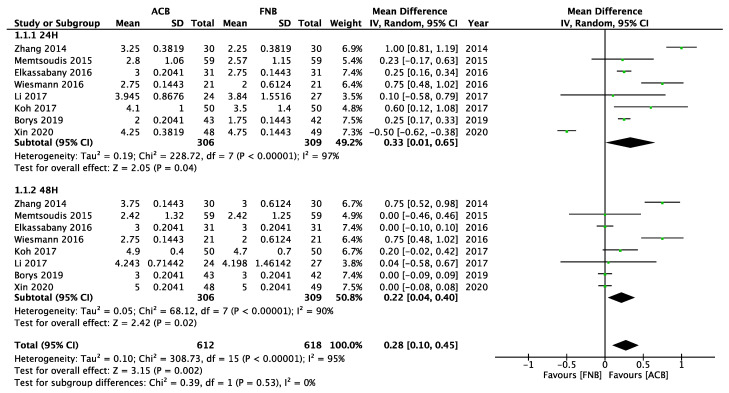
Forest plot of quadriceps muscle strength between the mixed ACB group and the FNB group “24 h, 48 h”.

Data from eight studies were pooled to evaluate the quadriceps muscle strength after receiving mixed ACB or FNB [17,78,79,89,100,101,131,141]. Among these eight studies, one study misinterprets the FTB as ACB [89]. Two studies used an undetermined definition of ACB [17,79]. FTB or proximal ACB cannot be determined because of the lack of ultrasound images or cited articles. The outcomes showed that the quadriceps muscle strength was more preserved in the mixed ACB group than in the FNB group in the first 24 and 48 postoperative hours. This result reached statistical significance but with high heterogeneity (Figure 5). The cephalad spreading of local anesthetics may anesthetize the motor fibers of the femoral nerve. Compared with FNB, ACB blocks the more distal site, and the motor-sparing effect becomes more significant. Further studies with a unified ACB technique are needed to explore the motor-sparing effect of ACB and FNB after knee surgery.

**Figure 6:**
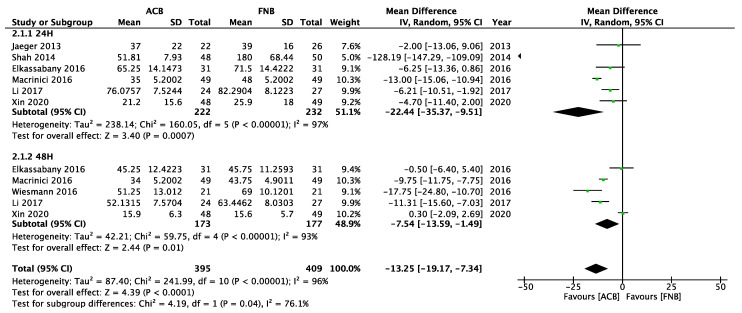
Forest plot of Time Up and Go test between the mixed ACB group and the FNB group (24 h, 48 h).

**Figure 7:**
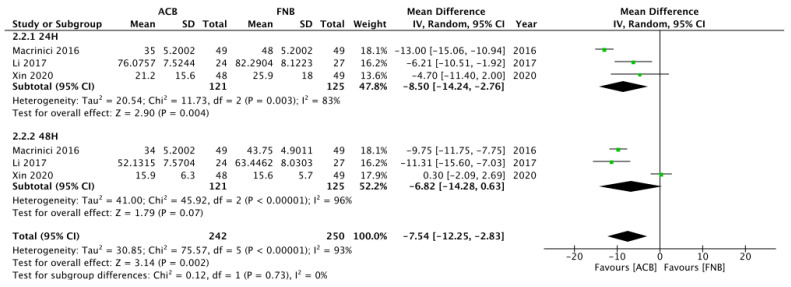
Forest plot of Time Up and Go test between “the ACB group with the exclusion of FTB and studies with undetermined definition” and the FNB group (24 h, 48 h).

The results of the mobilization ability between the mixed ACB group and the FNB group were available in six studies and were reported as Time Up and Go (TUG) tests (Figure 6). [17,32,34,93,101,131]. Among these six studies, two studies misinterpret the FTB as ACB [32,34]. One study used an undetermined definition of ACB [17]. FTB or proximal ACB cannot be determined because of the lack of ultrasound images or cited articles. After excluding the above three studies using the undetermined definition of ACB, we performed a subgroup analysis (Figure 7) [17,32,34]. The FNB group was inferior to the mixed ACB group or “the ACB group with the exclusion of FTB or studies with an undetermined definition” 24 and 48 h after surgery (Figure 6 and Figure 7), and this result was highly heterogeneous.

The analgesic effect between true ACB and FNB for knee surgery was insufficient to perform analysis [17]. Theoretically, placing the block more proximally may have an additional analgesic benefit [41]. The injection site and craniocaudal spread of the injectate were correlated with safety and efficacy. However, continuity between the AC and FT has been debated [37,38,39,142]. Clinically, a large volume of local anesthetic in the distal true AC made no difference in pain scores but resulted in quadriceps weakness (46 mL produced a 30% reduction in quadriceps strength following ACB in 50% of the patients, but without any complication on drop foot was mentioned in this study) [143]. Further well-designed studies, focusing on low volumes of local anesthetics and using the corrected classification system, are required to compare the analgesic effect between true ACB and FNB in major and minor knee surgeries, respectively.

### 3.2. The Effect of p-ACB versus d-ACB

In 2020, a meta-analysis comparing p-ACB with d-ACB for TKA concluded that both techniques had similar analgesic efficacy [144]. However, this result should be explained carefully due to a lack of disclosure of the exact injection level in all studies in regard to the adductor canal. Based on the corrected classification, insufficient studies were eligible to be pooled to evaluate the clinical effect of the proximal true ACB and the distal true ACB.

In our opinion, p-ACB is a better choice than d-ACB for a safe and effective block after knee surgery because of the nature of d-ACB, such as smaller branches and the variable position of involved nerves. The saphenous nerve consistently exists only in the proximal adductor canal (SN pierces VAM at mid-AC). These features make the clinical effect of d-ACB unpredictable. A letter from Gautier et al. described the occurrence of impaired dorsiflex and plantar flexion of the foot after d-ACB with 20 mL local anesthetics injected via the catheter [145]. The spread to the popliteal fossa with apparent contact with both divisions of the sciatic nerve was evidenced by the contrast medium. Although d-ACB may cover the posterior knee pain by blocking the sciatic and obturator nerves, it may also cause foot drop if the catheter tip is placed proximal to the adductor hiatus [145]. The most important way to achieve motor-sparing is to minimize injectate volume to prevent injectate from spreading to other unwanted targets. Therefore, choosing p-ACB allows ACB to be accomplished with the smallest volume, thus sparing motor function. Although current studies are insufficient to prove it, a more consistent conclusion will be achieved if this corrected classification system can be emphasized and applied in future studies.

### 3.3. The Effect of True ACB versus FTB

**Figure 8:**
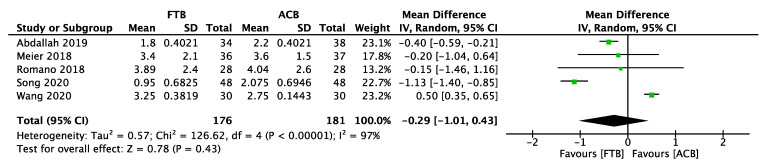
Forest plot of pain scores between the FTB group and the true ACB group (24 h).

**Figure 9:**
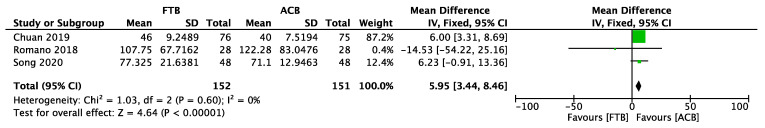
Forest plot of Time Up and Go test between the FTB group and the true ACB group (24 h).

Five studies compared pain scores between the FTB and the true ACB group 24 h after knee surgery [24,91,111,129,130]. No significant difference in pain scores 24 h after surgery was found between the two groups (Figure 8). Three studies compared TUG test scores 24 h after surgery between the two groups, and the true ACB group had preserved more mobilization ability (Figure 9) [24,129,142]. In these trials, they used inconsistent outcome measures, and quadriceps strength could not be analyzed. Pain scores from day 0 to day 1 between the two groups cannot be analyzed because of insufficient data. Further research comparing true ACB with FTB is needed to evaluate their motor-sparing effect and analgesic effects.

In brief, based on the corrected classification system, our meta-analysis can conclude that the quadriceps muscle strength and mobilization ability are more preserved in the mixed ACB group than the FNB group 24 h after knee surgery. Compared with the FTB group, the true ACB group preserves more mobilization ability 24 h after knee surgery. Still, more high-quality studies with large sample sizes are needed to explore the clinical effects of FTB, p-ACB, and d-ACB for knee surgeries in the future. The included studies had a low risk of bias after the assessment. Funnel-plot analyses were not performed because they should be used only when there are more than 10 studies included in the meta-analysis [146].

## 4. Other Considerations

### 4.1. Complications

Complications related to ACB may include injection pain, infection at the injection site, bleeding, nerve injury, allergic reaction, and local anesthetic systemic toxicity [147]. For ACB or FTB, there is a concern about an intravascular injection of local anesthetics because of the proximity of the femoral vessels [148]. With ultrasound guidance and a negative aspiration test, the risk of nerve injury, local toxicity, and vessel puncture can be reduced. Besides, the half-the-air setting can help identify the correct location using test volume injection other than local anesthetics and prevent incidental intraneural injection [149,150].

If local anesthetics spread to the proximal FT or popliteal fossa, it will block the femoral nerve or sciatic nerve, resulting in quadriceps weakness or foot drop [5,61,145,151]. Chen et al. described a case of quadriceps weakness after a mid-thigh injection of 20 mL of 0.5% ropivacaine [152]. Besides the injection pressure and anatomical variation, the volume may have contributed to the spread of the anesthetics to the proximal motor branches. An editorial commentary shared a case of delayed onset of foot drop after a true mid-AC injection via the catheter (initial injection of 20 mL of 0.2% ropivacaine, then 10-mL demand with 60-min lockout at 6 mL/h infusion). They proposed that the intercompartmental spread to the posterior compartment of the thigh muscle groups may have been created by the injection pressure [153]. Although true ACB is considered a sensory block, patients should be monitored for the occurrence of quadriceps weakness or foot drop. These complications will increase the risk of falls and delay functional recovery and rehabilitation.

### 4.2. Local Anesthetic Volume and Concentration

As of now, the optimal ACB regimen achieving adequate analgesia while avoiding side effects remains undefined. When reading relevant publications, we should be aware of the technique classified. A review article in 2016 reported that single-shot or catheter loading volumes of 15–30 mL at a ropivacaine concentration of 0.2% or 0.5% reveal no clinically significant influence on quadriceps strength [7]. This result should be taken cautiously because the authors mistook FTB as true ACB. Recently, a dose-finding study showed that the ED_50_ of 0.5% ropivacaine required to produce a 30% reduction in quadriceps strength in 50% of the patients was 46 mL when using sonographic landmarks to perform p-ACB [143]. In our clinical practice, injectate as low as 2–5 mL was enough to achieve adequate p-ACB.

Delayed quadriceps weakness has been reported in FTB using 8 mL/h of 0.2% ropivacaine of continuous infusion after TKA. In this case, contrast injection under fluoroscopy disclosed proximal spread approaching the common femoral nerve [151]. A recent multivariate analysis in 2020 revealed a 9% prevalence of quadriceps weakness after single-shot d-ACB (mean volume: 21.7 ± 5.3 mL). This study observed quadriceps weakness associated with females and incremental dosing of ACB volume per unit of body mass index (BMI) [154]. Higher BMI may prevent the spread of anesthetic agents to the FT in some manner. No studies have been conducted to determine the lowest effective concentration. According to the aforementioned evidence, a low volume of anesthetics is favored to prevent motor weakness.

### 4.3. Continuous versus Single-Shot ACB

The five latest meta-analyses comparing continuous mixed ACB with single-shot mixed ACB revealed that continuous mixed ACB provides better analgesia and results in a shorter length of hospital stay. This may not be a firm conclusion because of a lack of the use of a corrected classification system of ACB, insufficient data, small sample sizes of published trials, and high heterogeneity. Although continuous ACB can conquer the limitation of the short duration of single-shot ACB, it has introduced some disadvantages, such as technical difficulties, catheter obstruction, migration, leakage of local anesthetics, patient education needs, and infection. For carefully selected patients and experienced anesthesiologists, continuous ACB may be an effective postoperative pain management strategy [155,156,157,158,159].

### 4.4. Perineural Adjuvant of FTB

Numerous published RCTs showed the effect of adjuvants added to local anesthetics for ACB, such as dexamethasone, clonidine, buprenorphine, and dexmedetomidine. Most relevant studies actually used FTB as their ACB model. Adding 4 or 8 mg of dexamethasone to bupivacaine (0.25% or 0.5%) or 0.5% ropivacaine increased the duration of FTB by 5–8 h compared with placebo [110,117,160]. An adjunct of 0.50 μg/kg of dexmedetomidine prolongs the pain-free period by approximately 8 h compared with plain ropivacaine [99] and reduces the total consumption of morphine in the initial four-hour period [96]. An RCT involving healthy volunteers used clonidine as an adjuvant; however, clonidine (150 μg/mL) did not prolong the pain-free duration [97]. The addition of 200 μg of buprenorphine to 30 mL of 0.25% bupivacaine reduced the opioid consumption in the first 24 h after TKA [161]. Various adjuvants for prolonging peripheral nerve blocks have been studied, but the Food and Drug Administration has not yet approved any one of them. The following are some side effects and toxicity correlated with perineural adjuvants: pruritus, nausea and vomiting (opioid), hypotension (clonidine), and bradycardia (dexmedetomidine). Therefore, any perineural adjuvants should be used with caution and concern about side effects and potential toxicity [162].

### 4.5. ACB for Anterior Cruciate Ligament (ACL) Surgery

The best regional analgesic technique for patients undergoing ACL reconstruction (ACLR) remains controversial. Recent suggestions regarding ACB for ACL surgery in four systematic reviews and meta-analyses differ. Articles included in these meta-analyses had mixed ACB definitions, including FTB and proximal true ACB. Two meta-analyses reported that mixed ACB for pain control after ACLR is categorized as weak strength of evidence of benefit [163,164]. However, Sehmbi et al. found that mixed ACB provides modest analgesic benefits for minor arthroscopic knee surgery. The other two meta-analyses concluded that mixed ACB has an analgesic effect similar to that of FNB and spares more quadriceps muscle strength than FNB after ACLR [76,77]. However, these are not definitive conclusions because of the lack of using a corrected classification system, limited sample size, and heterogeneity, and most trials focused on pain severity instead of functional outcomes.

## 5. Conclusions

This review used a corrected classification system to categorize the FTB, p-ACB, and d-ACB. Several studies misinterpreting mid-thigh level injection as ACB should be corrected to FTB. After clarifying the anatomy and definition of these blocking techniques, the results of this subgroup meta-analysis showed that: (1) the mixed ACB group has a better motor-sparing effect (quadriceps muscle strength and TUG test) than the FNB group 24 h after knee surgery; (2) true ACB provides an analgesic effect similar to that of FTB and preserves more mobilization ability (TUG test) than FTB. FTB may be an alternative blocking method to FNB or ACB for analgesia after knee surgery. These clinical effects of the nerve blocks are also influenced by adjuvants, the volume and concentration of local anesthetics, and surgery. On postoperative day 0, FNB may be better than FTB or ACB because pain management is the major issue right after surgery. The well-explained risk of falls, careful patient education, and communication with the surgeon are essential before performing nerve blocks. On postoperative days 1–2, ACB is recommended for its motor-sparing effect during rehabilitation. Based on the accumulated evidence, physicians may consider all aforementioned factors to choose the most suitable nerve block technique for multimodal analgesic plans for patients undergoing knee surgery.

## Figures and Tables

**Table 1:** Summary of reported randomized control trials using different approach to the adductor canal block (total ACB related studies number = 130 ^#^).

Description Regarding the Needle (Catheter) Tip Position	Corrected Classification	Rationale	Number
Approach by surface landmarks			
Halfway between ASIS and patella	FTB	This position has been proven to be proximal to the adductor canal [36].	27
Halfway between greater trochanter to patella	ACB (not sure proximal or distal)	Adductor canal is located at the middle one-third of the distance from the base of the patella to the lower border of the greater trochanter [26].	4
Halfway between inguinal ligament and patella	ACB (not sure proximal or distal)	1. Adductor canal is located at the middle one-third of the distance from the base of the patella to the lower border of the greater trochanter [26]. 2. The lower border of greater trochanter approximates the level of inguinal crease [56].	12
Halfway between inguinal ligament and medial condyle	ACB (not sure proximal or distal)	1. Adductor canal is located at the middle one-third of the distance from the base of the patella to the lower border of the inguinal crease. 2. Medial condyles of femur articulate with the tibia and the patella. Medial condyles are distal than the base of the patella.	2
Approach by ultrasound guidance			
SFA covered by the medial border of sartorius muscles	FTB	Based on the representative image, the medial border of sartorius did not meet the medial border of adductor longus yet.	5
FA covered by the midpoint of sartorius muscles	FTB or proximal ACB	Whether the medial borders of sartorius and adductor longus meet could not be identified in the representative image.	4
FA underneath the sartorius muscles	FTB or ACB (not sure proximal or distal)	Depends on whether the medial borders of sartorius and adductor longus meet in the representative image.	10
Cavity surrounded by medial femoris, ALM, and sartorius muscles	FTB or proximal ACB	1. Depends on whether the medial borders of sartorius and adductor longus meet in the representative image. 2. Mid-adductor canal can be defined as ”distal to the proximal adductor canal, where the ALM is replaced by AMM posteromedially”.	5
2–3 cm proximal to femoral artery diving deep	Distal ACB	Using corrected classification system.	5
Numbers of inconsistent approaches of ACB related studies			66 *

^#^ Total 130 studies, 29 studies using corrected nomenclature, 35 studies cannot be defined due to unclear technique description, 66 studies using nonunified classification; * There are 3 studies comparing clinical effect of proximal, mid, and distal ACB; Abbreviations: adductor canal block (ACB), femoral triangle block (FTB), superficial femoral artery (SFA), anterior superior iliac spine (ASIS), adductor longus muscle (ALM), adductor magnus muscle (AMM).

**Table 2:** Corrected classification according to anatomical definition of the adductor canal.

Study	Original Grouping in the Study	Description Regarding the Needle (Catheter) Tip Position /Further Evidence in the Study	Corrected Classification /Rationale
Jæger 2013, 2014, 2018 [15,32,83]	ACB	Halfway between the ASIS and the patella; the catheter was advanced 1–2 cm beyond the tip of the needle.	FTB /This position has been proven to proximal to the adductor canal [36].
Kwofie 2013 [5]	ACB	The midpoint between the inguinal crease and the medial condyle. /Under ultrasound guidance, the femoral artery in short axis deep to the sartorius muscle can be visualized.	FTB or proximal ACB /The medial border of sartorius muscle didn’t meet the medial border of ALM in the representative image.
Hanson 2013, 2014 [84,85]	ACB	Mid-thigh, half the distance between the inguinal crease and the patella.	Proximal ACB /1. Adductor canal is located at the middle one-third of the distance from the base of the patella to the lower border of the greater trochanter [26]. 2. The lower border of greater trochanter approximates the level of inguinal crease. 3. FA is not diving in the representative image [56].
Grevstad 2014, 2015 [6,30]	ACB	Mid-thigh level, as described by Jæger 2012 [86] (with the FA, femoral vein, and the saphenous nerve deep to the sartorius muscle between the vastus medialis muscle and the ALM).	FTB or proximal ACB /No ultrasound image can be referred to. Whether the medial borders of sartorius and adductor longus meet could not be identified
Kim 2014 [87]	ACB	Mid-thigh level, as described by Manickam 2009 [13]. /By techniques of Manickam 2009, the injection site is 2–3 cm proximal to where the femoral artery diving deep.	Distal ACB /No ultrasound image can be referred to.
Mariano 2014 [88]	Proximal ACB	Where the medial border of sartorius first covers the superficial femoral artery; the catheter was advanced 1 to 3 cm beyond the needle tip posterior to the nerve.	FTB /1. Obviously, the medial border of sartorius did not meet the medial border of adductor longus yet. 2. The so-called distal ACB in the study should also be anatomically classified as FTB, not to mention the proximal one
	Distal ACB	Approximately halfway between the anterior superior iliac spine and the patella; the catheter was advanced 1 to 3 cm beyond the needle tip posterior to the nerve.	FTB /This position has been proven to be proximal to the adductor canal [36].
Shah 2014 [34]	ACB	Halfway between ASIS and the patella; a catheter was inserted 5–8 cm through the cannula.	FTB /This position has been proven to proximal to the adductor canal.
Zhang 2014 [89]	ACB	Approximately 8–12 cm below the inguinal crease; a catheter was placed 5 cm beyond the tip of the needle. /The authors used the definition by Ishiguro 2012, in the discussion section [90]. The site is where SFA passes beneath the medial border of the sartorius muscle.	FTB /Whether the medial borders of sartorius and adductor longus meet could not be identified in the representative image. By the Mariano 2014 [88] and Meier 2018 [91], the medial border of sartorius did not meet the medial border of adductor longus yet in this level.
Marian 2015 [92]	ACB	Mid-thigh level, the femoral artery was visualized in the short axis below the sartorius muscle.	FTB or proximal ACB /Whether the medial borders of sartorius and adductor longus meet could not be identified in the representative image.
Shah 2015 [35]	ACB	Halfway between the ASIS and the patella; a catheter was inserted 5–8 cm through the cannula.	FTB /This position has been proven to proximal to the adductor canal.
Abdallah 2016 [14]	ACB	Mid-thigh level. /The authors used the definitions by Manickam 2009 [13] in the method section. By techniques of Manickam 2009, the injection site is 2–3 cm proximal to where the femoral artery diving deep.	Distal ACB /No ultrasound image can be referred to.
Elkassabany 2016 [17]	ACB	Where the femoral artery was underneath the midpoint of the sartorius muscle with the vein just inferior to the artery; the catheter was advanced 2–3 cm beyond the tip of the needle. /The authors used the definitions by Ishiguro 2012 [90] and Mariano 2014 [88], but the injection site was more distal.	FTB or proximal ACB /There is no ultrasound image for reference.
Macrinici 2016 [93]	ACB	Where the femoral artery in short axis deep to the sartorius muscle. /The authors used the definitions by Manickam 2009 in the method section.	Distal ACB /There is no ultrasound image for reference. By techniques of Manickam 2009, the injection site is 2–3 cm proximal to where the femoral artery diving deep [13].
Messeha 2016 [94]	ACB	Mid-point between the inguinal crease and medial condyle.	ACB (not sure proximal or distal) /1. Adductor canal is located at the middle one-third of the distance from the base of the patella to the lower border of the inguinal crease. 2. Medial condyles of femur articulate with the tibia and the patella. Medial condyles are distal than the base of the patella.
Thapa 2016 [95,96]	ACB	Halfway between ASIS and the patella.	FTB /This position has been proven to be proximal to the adductor canal.
Wiesmann 2016 [79]	ACB	Where the femoral artery posterior to the sartorius muscle; the insertion point was 20 cm proximal to the cranial margin of the patella as measured by a ruler. A catheter wad inserted 3–5 cm over the needle.	FTB or proximal ACB /1. There is no ultrasound image or cited articles for reference. 2. Based on Wong 2016 [36], 20 cm proximal to the patella may be located in the femoral triangle.
Andersen 2017 [97,98]	ACB	Mid-thigh level, the femoral artery was visualized in the short axis below the sartorius muscle.	FTB or proximal ACB /There is no ultrasound image for reference.
Goyal 2017 [99]	ACB	Mid-thigh level. Use ultrasound proof to visualize sartorius muscle and femoral vessels. Target anterolateral to the femoral artery and below the sartorius.	FTB /From the ultrasound image, the medial border of sartorius muscle didn’t meet the medial border of ALM yet.
Koh 2017 [100]	ACB	Where the femoral artery was underneath the sartorius muscle; the needle was placed to the space bordered by vastus medialis, sartorius muscle, and femoral artery.	FTB or ACB (not sure proximal or distal) /No ultrasound image can be referred to. Whether the medial borders of sartorius and adductor longus meet could not be identified by the description.
Li 2017, 2020 [101,102]	ACB	Find a cavity surrounded by the sartorius muscle, medial femoral muscle, and the adductor muscles.	FTB or ACB (not sure proximal or distal) /There is no ultrasound image for reference. Whether the medial borders of sartorius and adductor longus meet could not be identified by the description.
Ghodki 2018 [103]	ACB	Halfway between ASIS and the patella.	FTB /This position has been proven to proximal to the adductor canal.
Grosso 2018 [104]	ACB	Halfway between ASIS and the patella.	FTB /This position has been proven to proximal to the adductor canal.
Kampitak 2018 [105]	ACB	Halfway between the inguinal crease and the patella.	ACB (not sure proximal or distal) /1. Adductor canal is located at the middle one-third of the distance from the base of the patella to the lower border of the inguinal crease. 2. Medial condyles of femur articulate with the tibia and the patella. Medial condyles are distal than the base of the patella.
Kampitak 2018 [106]	ACB	Mid-thigh level. /Based on the reference of Jaeger 2012 [86], Jenstrup 2012 [9], and Lund 2011 [8] from the introduction section, these articles defined mid-thigh level as halfway between ASIS and patella.	FTB /This position has been proven to proximal to the adductor canal.
Meier 2018 [91]	Proximal ACB	Where SFA existed underneath the medial third of sartorius (ultrasound image); a catheter was advanced 1 to 2 cm into the adductor canal. /The authors used the definitions by Mariano 2014 in the introduction section.	FTB /Obviously, the medial border of sartorius did not meet the medial border of adductor longus yet.
	Distal ACB	Where SFA was underneath the midpoint of the sartorius muscle.	FTB or proximal ACB /Whether the medial borders of sartorius and adductor longus meet could not be identified in the representative image.
Romano 2018 [24]	Proximal ACB	Where SFA passed beneath the medial border of sartorius. /The authors agreed with the definitions by Mariano 2014 and Meier 2018 when comparing the results of proximal and distal ACB in the discussion section.	FTB /The medial border of sartorius did not meet the medial border of adductor longus yet
	Distal ACB	Half of the distance between the inguinal crease and top of patella.	ACB (but not sure of proximal or distal) /1. Adductor canal is located at the middle one-third of the distance from the base of the patella to the lower border of the greater trochanter [26]. 2. The lower border of greater trochanter approximates the level of inguinal crease [56].
Rousseau-Saine 2018 [107]	ACB	Halfway between the ASIS and the patella.	FTB /This position has been proven to proximal to the adductor canal.
Runner 2018 [20]	ACB	The adductor canal was located by visualizing the FA on the short axis bordered by the sartorius muscle, the vastus medialis muscle, and the ALM.	FTB or proximal ACB /No ultrasound image can be referred to. Whether the medial borders of sartorius and adductor longus meet could not be identified.
Sztain 2018 [23]	Proximal ACB	Halfway between the ASIS and the patella.	FTB /This position has been proven to proximal to the adductor canal.
	Distal ACB	2–3 cm proximal to the adductor hiatus.	Distal ACB
Tong 2018 [108]	ACB	Mid-thigh approach based on Jæger 2013 [32], halfway between the ASIS and the patella.	FTB /This position has been proven to proximal to the adductor canal.
Turner 2018 [109]	ACB	Midpoint between the patella and inguinal crease.	ACB (but not sure of proximal or distal) /As explained above, adductor canal is located at the middle one-third of the distance from the patella and inguinal crease.
Turner 2018 [110]	ACB	Halfway between the ASIS and the patella.	FTB /This position has been proven to proximal to the adductor canal.
Abdallah 2019 [111]	Proximal ACB	Femoral artery medial to sartorius.	FTB /The medial border of sartorius did not reach the medial border of adductor longus in the representative image.
	Mid-ACB	Femoral artery inferior to sartorius.	FTB or proximal ACB /Whether the medial borders of sartorius and adductor longus meet could not be identified in the representative image.
	Distal ACB	Femoral artery lateral to sartorius.	Distal ACB /Femoral artery is diving in the representative image.
Canbek 2019 [112]	ACB	Mid-thigh level (halfway between the anterior superior iliac spine and the patella).	FTB /This position has been proven to proximal to the adductor canal.
Cicekci 2019 [113]	ACB	Midway between the inguinal ligament and the medial condyle. /An ultrasonographic image of the saphenous nerve was captured in the adductor canal, laterally to the femoral artery under the sartorius muscle.	ACB (not sure proximal or distal) /1. Adductor canal is located at the middle one-third of the distance from the base of the patella to the lower border of the inguinal crease. 2. Medial condyles of femur articulate with the tibia and the patella. Medial condyles are distal than the base of the patella.
Elkassabany 2019 [114]	ACB	Halfway between the ASIS and the patella.	FTB /This position has been proven to proximal to the adductor canal.
Faiaz 2019 [115]	ACB	Mid-thigh level. /Based on the citation of Jæger 2013 [4], mid-thigh level is at halfway between ASIS and the patella.	FTB /This position has been proven to proximal to the adductor canal.
Goytizolo 2019 [116]	ACB	Mid-thigh level. /The femoral artery was visualized under the sartorius muscle.	FTB or ACB (not sure proximal or distal) /No ultrasound image can be referred to. Whether the medial borders of sartorius and adductor longus meet could not be identified.
Ibrahim 2019 [117]	ACB	Midway between the ASIS and patella.	FTB /This position has been proven to proximal to the adductor canal.
Lim 2019 [21]	ACB	Mid-thigh, midway between the ASIS and patella.	FTB /This position has been proven to proximal to the adductor canal.
Lyngeraa 2019 [118]	ACB	Mid-thigh, midway between the ASIS and patella.	FTB /This position has been proven to proximal to the adductor canal.
Kastelik 2019 [119]	ACB	Identify the SFA below the sartorius muscle.	FTB or ACB (not sure proximal or distal) /No ultrasound image can be referred to. Whether the medial borders of sartorius and adductor longus meet could not be identified.
Kukreja 2019 [120]	ACB	Mid-thigh level. /Based on the citation of Jæger 2013 [32], mid-thigh level is at halfway between ASIS and the patella.	FTB /This position has been proven to proximal to the adductor canal.
Kulkarni 2019 [121]	ACB	Mid-thigh, halfway between the inguinal crease and patella.	ACB /1. Adductor canal is located at the middle one-third of the distance from the base of the patella to the lower border of the greater trochanter. 2. The lower border of greater trochanter approximates the level of inguinal crease.
Lan 2019 [122]	ACB	Mid-thigh level. /Based on the citation of Lund 2011 [8], mid-thigh level at halfway between ASIS and the patella.	FTB /This position has been proven to proximal to the adductor canal.
Lynch 2019 [123]	ACB	Target the saphenous nerve at the adductor hiatus.	Distal ACB
Stebler 2019 [124]	ACB	Mid-thigh site; target the triangular hyperechoic region lateral to the artery, bounded by the sartorius muscle superiorly, and the vastus medialis laterally.	FTB or ACB (not sure proximal or distal) /There is no ultrasound image for reference. Whether the medial borders of sartorius and adductor longus meet could not be identified from the paragraph.
Wang 2019 [125]	ACB	Half the distance between the inguinal crease and the patella.	ACB (but not sure of proximal or distal) /1. Adductor canal is located at the middle one-third of the distance from the base of the patella to the lower border of the greater trochanter. 2. The lower border of greater trochanter approximates the level of inguinal crease.
Gadsden 2020 [126]	ACB	The midpoint between the inguinal crease and the proximal aspect of patella.	ACB (but not sure of proximal or distal) /1. Adductor canal is located at the middle one-third of the distance from the base of the patella to the lower border of the greater trochanter. 2. The lower border of greater trochanter approximates the level of inguinal crease.
KAVAK AKELMA 2020 [127]	ACB	Halfway between the ASIS and the patella; visualize femoral artery under the sartorius muscle.	FTB /This position has been proven to proximal to the adductor canal.
Kertkiatkachorn 2020 [128]	ACB	Identify the sartorius muscle, ALM, vastus medialis muscles, and the femoral artery. Position the needle tip between the superficial femoral artery and the posterior region of the sartorius muscle.	FTB or proximal ACB /1. No sonographic image can be referred to. Whether the medial borders of sartorius and adductor longus meet cannot be identified. 2. Mid-adductor canal can be defined as “distal to the proximal adductor canal, where the ALM is replaced by AMM posteromedially”.
Song 2020 [129]	FTB	5 cm proximal to the apex of femoral triangle, identified as the intersection of the medial borders of the sartorius and adductor longus muscles.	FTB
	ACB	5 cm distal to the apex of femoral triangle.	Almost mid-adductor ACB /1. The femoral artery was underneath the sartorius muscle, not diving deep in the representative image. 2. Mid-adductor canal can be defined as ”distal to the proximal adductor canal, where the ALM is replaced by AMM posteromedially”. The ALM was almost replaced by AMM from the representative image.
Wang 2020 [130]	FTB	Halfway between the ASIS and the base of the patella; a catheter was inserted 3 cm past the needle tip.	FTB
	ACB	The midpoint between the greater trochanter of the femur and the base of the patella.	Proximal ACB /1. Adductor canal surrounded by the sartorius muscle, the vastus medialis muscle, and the adductor longus muscle was identified clearly from the representative image. 2. Adductor canal is located at the middle one-third of the distance from the base of the patella to the lower border of the greater trochanter [26].
Xin 2020 [131]	ACB	The probe was placed on the middle of the thigh. A catheter was inserted 3 cm past the needle tip. /The authors use the techniques described by Manickam 2009.	Distal ACB /There is no ultrasound image for reference. By techniques of Manickam 2009, the injection site is 2–3 cm proximal to where the femoral artery diving deep [13].
Greenky 2020 [132]	ACB	The probe was placed anteromedially on the middle and distal third of the thigh. /The femoral artery and sartorius muscle formed the roof of the adductor canal.	FTB or proximal ACB /Whether the medial borders of sartorius and adductor longus meet cannot be identified from the sonographic image.
Kampitak 2020 [133]	ACB	Halfway between the ASIS and the base of the patella.	FTB
Raddaoui 2020 [134]	ACB	The probe was positioned transversely midway from the patella and the inguinal ligament.	ACB /1. Adductor canal is located at the middle one-third of the distance from the base of the patella to the lower border of the greater trochanter. 2. The lower border of greater trochanter approximates the level of inguinal crease.
Mittal 2021 [135]	ACB	Halfway between the ASIS and the base of the patella.	FTB
Rambhia 2021 [136]	ACB	The adductor canal catheter was placed as per our standard technique, at the mid-point between the inguinal crease and the superior pole of the patella.	ACB /1. Adductor canal is located at the middle one-third of the distance from the base of the patella to the lower border of the greater trochanter. 2. The lower border of greater trochanter approximates the level of inguinal crease.
Saini 2021 [137]	ACB	Probe was placed transversely on medial aspect of mid-thigh; midway between ASIS and base of patella.	FTB
Wang 2021 [138]	ACB	Probe was placed on the anteromedial part of the thigh at the level of the midpoint, between the greater trochanter of the femur and the proximal edge of patella.	ACB /Adductor canal is located at the middle one-third of the distance from the base of the patella to the lower border of the greater trochanter.
Wang 2021 [139]	ACB	Probe was used to scan the middle of the thigh (half the distance between the inguinal crease and the patella)	ACB /1. Adductor canal is located at the middle one-third of the distance from the base of the patella to the lower border of the greater trochanter. 2. The lower border of greater trochanter approximates the level of inguinal crease.
Zheng 2022 [140]	ACB	Probe was placed at the midpoint of the connecting line between the greater trochanter of the femur and the upper edge of the patella.	ACB /Adductor canal is located at the middle one-third of the distance from the base of the patella to the lower border of the greater trochanter.

Abbreviations: adductor canal block (ACB), femoral triangle block (FTB), superficial femoral artery (SFA), descending genicular artery (DGA), anterior superior iliac spine (ASIS), adductor longus muscle (ALM), adductor magnus muscle (AMM).

## Data Availability

Not applicable.
